# Nigerian Hospital and Community Pharmacists’ Knowledge, Awareness, and Perceptions of Autism Spectrum Disorders

**DOI:** 10.1177/11786329241299314

**Published:** 2024-11-12

**Authors:** Ayobami Adesoji Aiyeolemi, Ogochukwu Ukamaka Amaeze, Veronica Okugbeni, Oyinlade Kehinde, Adekunle Faid Adeleke, Jamie C Barner

**Affiliations:** 1 Health Outcomes Division, College of Pharmacy, University of Texas, Austin, TX, USA; 2 Department of Clinical Pharmacy & Biopharmacy, Faculty of Pharmacy, University of Lagos, Nigeria; 3 Federal Neuro-psychiatric Hospital, Yaba, Lagos, Nigeria

**Keywords:** Autism spectrum disorders, knowledge, awareness, perceptions, pharmacists, Nigeria

## Abstract

**Background:**

Pharmacists can play a role in enhancing treatment outcomes of autistic people, but they must possess sufficient knowledge and awareness of autism spectrum disorders (ASD). Current evidence is scant among Nigerian pharmacists. The objectives of this study were to: (1) Describe and compare Nigerian hospital and community pharmacists’ ASD knowledge, awareness, and perceptions; (2) Determine if there is a significant correlation between ASD knowledge, awareness, and perceptions; (3) Determine if demographic and practice-related factors are significantly related to pharmacists’ ASD awareness.

**Method:**

Hospital and community pharmacists were administered a self-report survey to assess ASD knowledge and awareness, as well as confidence in caring for autistic people, and perceived benefits of ASD training and care. Data were collected from August to December 2021 and analyzed using inferential and descriptive statistics. Cronbach’s alphas were used to assess reliability.

**Results:**

Of respondents, (Total N = 383; N = 201 hospital pharmacists from various states) and N = 182 community pharmacists in Lagos state) community pharmacists had significantly higher mean knowledge than hospital pharmacists (58.10% ± 19.00% vs 53.20% ± 20.10%; *P* = .016). Overall mean awareness score was 2.90 ± 0.80, with no significant difference between community and hospital pharmacists (*P* = .096). Perception regarding ASD continuing education (CE) was strongly correlated with the perceived benefits of pharmacy services to autistic people in Nigeria (*r* = .69; *P* < .0001). Additionally, awareness was positively correlated with knowledge (*r* = .47, *P* < .0001) and perceived confidence in ASD medication counseling (*r* = .54, *P* < .0001). Multivariate analysis revealed that being a hospital pharmacist, having courses on ASD during pharmacy degree programs, undergoing ASD CE, and having <5 years of practice experience were significantly (*P* < 0.05) associated with higher ASD awareness.

**Conclusion:**

Respondents had suboptimal knowledge and awareness of ASD. Including ASD in the pharmacy curriculum and providing CE programs may help improve pharmacists’ ability to provide more optimal patient care services for autistic people.

## Introduction

Autism spectrum disorders (ASD) constitute a group of intricate neurodevelopmental disorders that typically emerge early in childhood.^1,2^ Autistic people typically have persistent limitations in social interactions and verbal and nonverbal communication, and they can exhibit highly restricted or repetitive behaviors.^3^ ASD are also often associated with other behavioral symptoms, including hyperactivity, anxiety, tantrums, self-harm, impulsivity, distractibility, and aggressiveness.^4,5^ The cause of ASD is unknown; however, it is thought to occur due to a multifaceted interplay of genetic and environmental influences.^6^ Globally, prevalence is estimated at 52 million, with 1% to 2% being children.^7^ In Nigeria, it is estimated that 1 in every 150 to 160 children (~600 000) has an ASD.^8^ Over the past 30 years, ASD prevalence has increased steadily globally.^9-14^ Several factors, including increased awareness and changing diagnostic criteria, have contributed to the increasing prevalence of ASD.^9-14^ Nonetheless, ASD is a global public health concern requiring concerted healthcare efforts.

Autistic people often have comorbid psychiatric and medical conditions, including depression, attention deficit hyperactivity disorder, sleep disorders, and seizures at rates exceeding the general population.^15-24^ Given the diversity of symptoms and comorbidities associated with ASD, autistic people are typically prescribed multiple medications^25-28^ and may sometimes react paradoxically to them.^26,29-31^ Risperidone and aripiprazole are the only medications currently approved by the Food and Drug Administration (FDA) to treat behavioral symptoms of ASD^32-34^; however, other psychotropic medications such as antidepressants, antipsychotics, stimulants, and anxiolytics have also been used, as well as complementary and alternative treatments.^28,35^ Due to complex medication use in autistic people, pharmacists can be instrumental in optimizing medications.

Pharmacists in Nigeria provide a range of healthcare services, including compounding and dispensing medications, patient counseling and education, treating mild ailments, point-of-care testing, and vaccinations.^36-38^ Having an optimal understanding of ASD enables pharmacists to communicate effectively with autistic people and their caregivers to ensure appropriate medication use and optimal outcomes.^39^ Furthermore, pharmacists’ recognition of ASD symptoms can help make pharmacies more accessible to autistic people. For example, minimizing visual and auditory triggers when consulting with autistic people.^39,40^ They could also refer individuals exhibiting ASD symptoms to relevant institutions for optimal care.^26^ In Nigeria and other Sub-Saharan African countries, pharmacological interventions are used more often than behavioral interventions.^41^ Thus, pharmacists are typically more involved in the care process and, therefore, need to be knowledgeable regarding ASD. While current research is limited, previous studies have revealed gaps in pharmacists’^26,40,42-44^ and pharmacy students’^25^ knowledge of the etiology, symptoms, and pharmacotherapy of ASD. One study found that over 40% of pharmacists did not know about FDA-approved medications for treating ASD.^42^ Another study found that almost 30% of pharmacists had the misconception that ASD is a precursor for schizophrenia.^43^ In Nigeria, previous studies have revealed low to moderate levels of ASD knowledge among nurses,^9,45-48^ physicians,^9,49^ and medical students.^50,51^ However, to the authors’ knowledge, there is no data on Nigerian pharmacists’ knowledge and awareness of ASD.

This study aimed to assess Nigerian hospital and community pharmacists’ knowledge, awareness, and perceptions regarding ASD. Specific objectives are: (1) To describe and compare Nigerian hospital and community pharmacists’ knowledge, awareness, and perceptions of ASD; (2) To determine if there is a significant correlation between pharmacists’ knowledge, awareness, and perceptions of ASD; (3) To determine if demographic and practice-related factors are significantly related to pharmacists’ awareness of ASD.

## Methods

### Participants, data collection, and analysis

This study was a descriptive cross-sectional self-administered survey of practicing hospital and community pharmacists in Nigeria. To be included, participants had to be fully licensed pharmacists practicing in either community or hospital pharmacy settings. Non-pharmacists and pharmacists practicing in other settings outside of hospital and community were excluded. The hospital pharmacist sample was recruited from individuals attending the 22nd Annual Scientific Conference of the Association of Hospital and Administrative Pharmacists of Nigeria (AHAPN) in August 2021. The AHAPN conference is Nigeria’s largest meeting of hospital and administrative pharmacists, with an estimated 400 hospital pharmacists in attendance. Meeting participants were approached (N = 240) and invited to participate in the study. For the community pharmacist sample, invitations to participate in the online survey were sent to the WhatsApp group platforms of the 24 administrative zones of the Lagos State Chapter of the Association of Community Pharmacists of Nigeria from September to December 2021.

A convenience sample (N = 240) of hospital pharmacists attending the conference were approached and invited to participate in the study. Those who met the inclusion criteria were given the self-administered questionnaire after consenting to participate in the study. Surveys were completed anonymously and returned to the researchers. For the community pharmacist sample, invitations were sent via the WhatsApp platform by zonal administrative heads on a weekly basis for 10 weeks to remind pharmacists to participate in the study. The total sample of community pharmacists invited is unknown due to uncertainty with online listserv membership and responsiveness. To avoid duplicate responses, hospital pharmacists at the conference were asked to respond to the survey only once, and the web-based survey used for community pharmacists was configured to allow one response per electronic device.

Descriptive statistics (means, standard deviation, frequencies, and percentages) were conducted for all study variables. Independent samples *t*-tests were used to compare knowledge, awareness, and perceptions between hospital and community pharmacists. Pearson’s correlations were used to assess the correlation between pharmacists’ knowledge, awareness, and perceptions of ASD. Multivariate linear regression was used to assess demographic and practice-related factors associated with pharmacists’ awareness of ASD. Internal consistency of the adapted scales was assessed using Cronbach’s alpha. Statistical significance was set at *P* < .05, and analyses were conducted using SAS 9.4 (SAS Institute, Cary, N.C). This study was approved by the Health and Research Ethics Committee of Lagos University Teaching Hospital (approval number: ADM/DSCST/HREC/APP/4476). All participants provided their written informed consent.

### Survey instrument

Primary outcomes were knowledge, awareness, and perceptions of ASD. Knowledge of ASD was assessed using an adapted scale (Cronbach’s alpha = .68)^52^ consisting of 12 items, which assessed pharmacists’ knowledge of the etiology, prevalence, pharmacotherapy, and symptoms of ASD.^25,26,42,52^ Response categories included true, false, and don’t know. Respondents received one point for correct responses and zero points for incorrect or don’t know responses. The points were summed and divided by the total number of questions for a percentage correct score (range 0%-100%). Higher scores correspond to higher knowledge of ASD. Awareness of ASD was measured using a previous unvalidated scale^25,26,42^ consisting of 6 items that assessed pharmacists’ familiarity with symptoms, medications used to manage ASD, and assisting families of autistic people to make informed decisions. Items were measured using a 5-point scale ranging from 1 = not familiar at all to 5 = completely familiar. Higher scores on the scale correspond to higher awareness of ASD. To measure perceptions, 5 single items were used. One item was created by the authors of the current study, and 4 items were adapted from previous studies.^26,42^ The items adapted from previous studies are unvalidated. The items assessed pharmacists’ confidence in caring for people with ASD, perceptions of the benefits of incorporating ASD courses in pharmacy education, undergoing continuing education (CE) programs on ASD, and benefits of pharmacy services to autistic people in Nigeria. All 5 single-items were measured using a 5-point scale ranging from 1 = strongly disagree to 5 = strongly agree, with higher scores corresponding to more positive perceptions. Demographics (age, gender, education) and practice-related characteristics (number of years of practice) were also assessed. Additionally, pharmacists were asked if they had lectures on ASD during their pharmacy degree program and if they had undergone CE programs on ASD. The survey was pretested separately with 2 groups of pharmacists, hospital and community (N = 23 each), to ensure clarity and readability. In response to feedback from hospital pharmacists, a “don’t know” option was added to the knowledge scale and incorporated in the final study survey. The survey was administered in English, which is Nigeria’s official language. See Supplemental File for the complete study instrument.

## Results

A total of 240 surveys were distributed to hospital pharmacists at the conference, of which 212 were returned, yielding a response rate of 88.3%. Among the 212 returned surveys, 11 were excluded due to missing data, resulting in 201 completed questionnaires (response rate of 83.7%). Additionally, 182 community pharmacists from 18 of the 24 administrative zones of the Lagos State chapter of the ACPN responded to the survey. As the total number of community pharmacists on the listserv could not be determined, a response rate for this group could not be calculated. In total, 383 responses were included in the analysis.

### Demographic and practice-related factors

Among *hospital pharmacists*, 38.3% were aged between 46 and 55 years, with over one-half (56.7%) being male and 36.8% having >20 years of practice experience. The majority (87.1%) did not receive lectures on ASD during their pharmacy program and had not participated in any CE programs on ASD (88.6%). Among *community pharmacists*, 43.4% were less than 35 years old, approximately half (50.5%) were male, and 28.6% had 5 to 10 years of practice experience. Over three-quarters (78.0%) did not receive lectures on ASD during their pharmacy program. Additionally, most respondents (91.2%) had not participated in any CE program on ASD. Compared to hospital pharmacists, community pharmacists were significantly (*P* < .05) younger, were less likely to have advanced degrees, had been in practice for shorter periods of time, and were more likely to have had ASD training in pharmacy school (Table 1).

**Table 1.** Comparison of respondent demographic and practice-related factors (N = 383).

Factor	Frequency (%)		*P*-value^a^
	Hospital (N = 201)	Community (N = 182)	
Age			<.001
<35	35 (17.4)	79 (43.4)	
36-45	60 (29.9)	42 (23.1)	
46-55	77 (38.3)	39 (21.4)	
⩾55	28 (13.9)	22 (12.1)	
Missing	1 (0.5)	0 (0.0)	
Gender			.227
Male	114 (56.7)	92 (50.5)	
Female	87 (43.3)	90 (49.5)	
Highest educational/professional qualification			<.001
Bachelor of Pharmacy	81 (40.3)	136 (74.7)	
Advanced degrees (Master’s, PharmD, Fellowship)	120 (59.7)	46 (25.3)	
Number of years of practice			< .001
<5	15 (7.5)	39 (21.4)	
5-10	44 (21.9)	52 (28.6)	
11-15	37 (18.4)	22 (12.1)	
16-20	30 (14.9)	22 (12.1)	
>20	74 (36.8)	47 (25.8)	
Missing	1 (0.5)	0 (0.0)	
ASD training in pharmacy school			.019
Yes	26 (12.9)	40 (22.0)	
No	175 (87.1)	142 (78.0)	
ASD continuing education			.392
Yes	23 (11.4)	16 (8.8)	
No	178 (88.6)	166 (91.2)	

Abbreviation: ASD: autism spectrum disorder.

Missing values were deleted for bivariate analysis.

a Chi-square; *P* < .05 denotes significant difference.

### Description and comparison of pharmacists*’* knowledge, awareness, and perceptions of ASD

The overall mean knowledge score (hospital and community pharmacists) was 55.50% ± 19.70% (scale range: 0%-100%), which shows that knowledge was suboptimal. Respondents exhibited the highest level of knowledge regarding social and behavioral impairments associated with ASD (96.1%), as well as recognizing that ASD is a developmental disorder (82.0%). Conversely, respondents were least knowledgeable about the gender prevalence of ASD (25.3%), prevalence of ASD compared to Down syndrome (32.4%) and juvenile diabetes (36.5%), as well as the non-rarity of ASD (33.7%). Notably, almost half of hospital (43.3.%) and community (46.7%) pharmacists responded “don’t know” when asked if vaccines could cause ASD. Also, more than half of the hospital (50.3%) and community (56.6%) pharmacists responded “don’t know” when asked if risperidone and aripiprazole are approved by the FDA for treatment for ASD. An independent t-test showed that the mean knowledge score of community pharmacists (58.10% ± 19.00%) was significantly higher than that of hospital pharmacists (53.20% ± 20.10%) (*P* = .016). Hospital and community pharmacists’ responses differed significantly on 4 of the 12 items. Community pharmacists demonstrated greater knowledge than hospital pharmacists in recognizing ASD as developmental disorders (*P* = .038), understanding the social and behavioral impairments associated with ASD (*P* = .029), and knowing that emotionally distant and rejecting parents do not cause ASD (*P* = .011), as well as in recognizing that ASD are not rare disorders (*P* = .036). The 12-item knowledge scale showed acceptable reliability with a Cronbach’s alpha of .75 (Table 2).

**Table 2.** Chi-square and *t*-test comparison of ASD knowledge^a^ between hospital and community pharmacists (N = 383).

Items	Correct response	Overall N = 383	Hospital N = 201	Community N = 182	*P*-value^c^
	T/F^b^	N (% Correct)	N (% Correct)	N (% Correct)	
ASD is a developmental disorder	T	314 (82.0)	157 (78.1)	157 (**86.3**)	.038
Children with ASD have impairments in social interaction, communication or language, and behavioral development	T	368 (96.1)	189 (94.0)	179 (**98.4**)	.029
ASD occurs more commonly among males than females	T	97 (25.3)	47 (23.4)	50 (27.5)	.358
ASD is more prevalent than juvenile diabetes	T	140 (36.5)	68 (33.8)	73 (40.1)	.169
ASD is more prevalent than Down syndrome	T	124 (32.4)	66 (32.8)	58 (31.9)	.840
ASD is curable	F	198 (51.7)	95 (47.3)	103 (56.6)	.068
Risperidone and aripiprazole have been approved by the FDA for the treatment of irritability associated with ASD	T	170 (44.4)	92 (45.8)	78 (42.9)	.567
Vaccines can cause ASD	F	178 (46.5)	98 (48.8)	80 (44.0)	.350
ASD exists only in childhood	F	240 (62.7)	117 (58.2)	123 (67.6)	.058
ASD is caused because of emotionally distant rejecting parents	F	289 (75.5)	141 (70.2)	148 (81.3)	.011
Genetic factors play a major role in the etiology of ASD	T	304 (79.4)	156 (77.6)	148 (81.3)	.371
ASD is a rare disorder	F	129 (33.7)	58 (28.9)	71 (39.0)	.036
Overall Knowledge (mean (SD))^d^	-	55.50 (19.70)	53.20 (20.10)	58.10 (19.00)	.016^e^

Abbreviations: ASD, autism spectrum disorder; FDA, United States Food and Drug Administration.

a Knowledge score range from 0% to 100%.

b F, false; T, true.

c Chi-square; *P* < .05 denotes significant difference.

d Cronbach’s alpha = 0.75.

e T-test; *P* < .05 denotes significant difference.

Table 3 shows respondents’ awareness of symptoms, treatment, and community resources for ASD care. The overall mean awareness score (hospital and community pharmacists) was 2.90 ± 0.80. While respondents exhibited the highest level of awareness (i.e., familiarity) concerning the classes of medications used in ASD treatment (3.30 ± 1.10) and the side effects of these medications (3.30 ± 1.10), the mean scores showed that they were only moderately familiar. Conversely, respondents displayed the least awareness regarding community resources available in their region for the referral of children exhibiting ASD symptoms (2.20 ± 1.10). There was no significant difference in mean awareness of hospital pharmacists (3.00 ± 0.80) and community pharmacists (2.90 ± 0.80) (*P* = .096). However, hospital pharmacists (2.30 ± 1.00) were more familiar with community resources for referring a child with ASD symptoms than community pharmacists (2.10 ± 1.1) (*P* = .014) although this may not be practically significant. Cronbach’s alpha for the awareness scale was .85, signifying acceptable reliability.

**Table 3.** *T*-test comparison of mean ASD awareness^a^ between hospital and community pharmacists (N = 383).

Items Are you familiar with. . .	Overall N = 383	Hospital N = 201	Community N = 182	*P*-value^b^
	Mean (SD)	Mean (SD)	Mean (SD)	
The different symptoms of ASD?	2.90 (1.00)	2.90 (1.00)	2.90 (1.00)	.831
The specific behaviors associated with ASD that medications seek to alleviate (eg, hyperactivity, obsessive compulsive disorder, and self-injury)?	3.10 (1.10)	3.20 (1.00)	3.10 (1.10)	.457
The different classes of medications (eg, antipsychotics, antidepressants, stimulants) that are used in treating the symptoms of ASD?	3.30 (1.10)	3.30 (1.00)	3.20 (1.20)	.281
The various side effects produced by medications used in the treatment of ASD symptoms (eg, sedation, irritation, extrapyramidal symptoms)?	3.30 (1.10)	3.30 (1.00)	3.20 (1.20)	.490
How to help families sort through information to make informed decisions about their child with ASD?	2.70 (1.10)	2.80 (1.00)	2.60 (1.10)	.054
Community resources in your region that can be used for referral of a child who is exhibiting symptoms commonly associated with ASD?	2.20 (1.10)	2.30 (1.00)	2.10 (1.10)	.014
Overall awareness^c^	2.90 (0.80)	3.00 (0.80)	2.90 (0.80)	.096

Abbreviation: ASD, autism spectrum disorder.

a Awareness: 1 = not familiar at all; 5 = completely familiar.

b *P* < .05 denotes significant differences.

c Scale Cronbach’s alpha = .85.

Regarding perceptions of ASD (Table 4), respondents were neutral about their confidence in providing medication counseling to parents of autistic people (3.20 ± 1.20), as well as their comfort levels in dispensing medications used to treat ASD (3.60 ± 1.10). However, the respondents strongly agreed that they would benefit from CE on ASD (4.60 ± 0.80) and that the pharmacy curriculum should incorporate courses on ASD (4.60 ± 0.80). Additionally, respondents indicated strong agreement that autistic people in Nigeria could benefit from services provided by pharmacists (4.70 ± 0.80). When comparing perceptions of ASD, respondents who were hospital pharmacists reported significantly higher confidence in their ability to provide ASD medication counseling (3.40 ± 1.10) compared to those who were community pharmacists (3.10 ± 1.20) (*P* = .003). Additionally, respondents who were hospital pharmacists reported significantly higher comfort levels with dispensing medications used to treat ASD (3.70 ± 1.00) compared to those who were community pharmacists (3.40 ± 1.10) (*P* = .008).

**Table 4.** *T*-test comparison of mean ASD perceptions^a^ between hospital and community pharmacists (N = 383).

Items I feel. . .	Overall N = 383	Hospital N = 201	Community N = 182	*P*-value^b^
	Mean (SD)	Mean (SD)	Mean (SD)	
Confident in my ability to counsel parents about the medication profile and side effects of prescriptions being used for the treatment of their child with ASD	3.20 (1.20)	3.40 (1.10)	3.10 (1.20)	.003
Comfortable dispensing medications used in the treatment of ASD	3.60 (1.10)	3.70 (1.00)	3.40 (1.10)	.008
That I could benefit from taking a CE or training program in the area of ASD	4.60 (0.80)	4.60 (0.80)	4.70 (0.80)	.300
That pharmacy school curriculum should include a course or lecture in the area of ASD	4.60 (0.80)	4.50 (0.80)	4.60 (0.80)	.183
That autistic people in Nigeria could benefit from pharmaceutical care services offered by pharmacists	4.70 (0.80)	4.70 (0.70)	4.70 (0.80)	.878

Abbreviation: ASD, autism spectrum disorder.

a Perception: 1 = Strongly disagree; 5 = Strongly agree.

b *P* < .05 denotes significant differences.

### Correlation between pharmacists’ knowledge, awareness, and perceptions of ASD

Seventeen of the 21 Pearson correlation analyses were significant and positive (*P* < .05). Among these, 6 were notably strong, including between: perceptions regarding benefits of ASD CE and benefits of pharmacy services to autistic people in Nigeria (*r* = .69; *P* < .0001); perceptions regarding including ASD courses in pharmacy school curriculum and benefits of pharmacy services to autistic people in Nigeria (*r* = .65; *P* < .0001); confidence in providing ASD medication counseling and comfortability with dispensing medications used to treat ASD (*r* = .65; *P* < .0001); perceptions regarding benefits of ASD CE and perceptions regarding including ASD courses in pharmacy curriculum (*r* = .64, *P* < .0001); awareness of ASD and confidence in providing ASD medication counseling (*r* = .54, *P* < .0001); and awareness of ASD and knowledge of ASD (*r* = .47, *P* < .0001) (Table 5).

**Table 5.** Pearson’s correlation of pharmacists’ knowledge, awareness, and perceptions regarding ASD.

ASD Variable	Knowledge	Awareness	Counseling	Dispensing	ASD CE	ASD courses	Benefits
Knowledge							
*r*	1.00	.47*	.23*	.18*	.11*	.11*	.15*
*P*-value		<0.0001	<0.0001	0.001	0.034	0.033	0.002
*N*	383	381	383	383	382	383	383
Awareness							
*r*		1.00	.54*	.44*	.13*	.16*	.18*
*P*-value			<0.0001	<0.0001	0.014	0.002	0.000
*N*		381	381	381	380	381	381
Counseling							
*r*			1.00	.65*	0.07	0.10	0.10
*P*-value				<0.0001	0.191	0.058	0.054
*N*			383	383	382	383	383
Dispensing							
*r*				1.00	.14*	0.07	.18*
*P*-value					0.005	0.145	0.001
*N*				383	382	383	383
ASD CE							
*r*					1.00	.64*	.69*
*P*-value						<0.0001	<0.0001
*N*					382	382	382
ASD courses							
*r*						1.00	.65*
*P*-value							<0.0001
*N*						383	383
Benefits							
*r*							1.00
*P*-value							
*N*							383

Abbreviations: ASD = autism spectrum disorder; ASD CE = perceptions regarding benefits of ASD continuing education programs; ASD courses = perceptions regarding including ASD courses in pharmacy school curriculum; Awareness = ASD awareness; Benefits = Perceptions regarding the benefits of pharmacy services to autistic people in Nigeria; Counseling = confidence in providing ASD medication counselling; Dispensing = comfortability with dispensing medications used to treat ASD; Knowledge = ASD knowledge.

Bold indicates strong correlations (⩾~0.5).

* Indicates significance at *P* < .05.

### Demographic and practice-related factors related to pharmacists’ awareness of ASD

To address Objective 3, a regression was conducted to determine demographic and practice factors related to pharmacists’ awareness of ASD. Some variables were excluded from the regression model due to multicollinearity. In the final model (Table 6), the following were positively and significantly (*P* < .05) associated with awareness: being a hospital pharmacist, and having: less than 5 years of practice experience; higher ASD knowledge scores; ASD training during pharmacy school; CE on ASD; higher confidence in providing ASD medication counseling; and higher perceptions regarding the benefit of pharmacy services to autistic people in Nigeria. The regression model was significant (*P* < .0001), and it explained a considerable amount of variance in awareness (adjusted *R*^2^ = .48).

**Table 6.** Multiple regression of factors related to pharmacist’ awareness of ASD (N = 383).

Factors	Parameter estimate (β)	*t*	*P*-value
Male	0.004	0.06	.950
Hospital pharmacist	0.170	2.54	.015
Higher degrees (Master’s, PharmD, Fellowship)	–0.013	–0.20	.843
Number of years of practice			
<5	0.270	2.65	.008
6 to 10	0.098	1.18	.240
11 to 15	–0.018	–0.19	.852
16 to 20	0.031	–0.32	.752
Knowledge score	0.013	8.21	<.0001
ASD training in pharmacy school			
(Yes)	0.280	3.35	.001
CE program on ASD (Yes)	0.490	4.72	<.0001
Perceived confidence in providing medication counseling to parents of autistic people	0.220	6.31	<.0001
Perceived level of comfort with dispensing ASD medications	0.068	1.80	.073
Perceived benefit of pharmacy services to autistic people in Nigeria	0.100	2.42	.016

Abbreviations: ASD, autism spectrum disorders; CE, continuing education.

*F* statistic = 28.01, Adjusted *R*^2^ = .48, *P* < .0001.

*P* < .05 denotes significant differences.

Reference categories: female, community pharmacist, Bachelor of Pharmacy, >20 years of practice, no.

## Discussion

This study aimed to assess and compare knowledge, awareness, and perceptions regarding ASD among hospital and community pharmacists in Nigeria. Our findings revealed gaps in hospital and community pharmacists’ knowledge and awareness of ASD, as well as their confidence in providing ASD medication counseling and comfortability with dispensing medications used to treat ASD. ASD prevalence has increased steadily over the last 3 decades, and this trend is expected to continue in the future.^12-14^ A recent study found that global prevalence increased from 20 million in 1990 to over 28 million in 2019.^13^ Because of the complex and multiple medication regimens, pharmacists who possess adequate knowledge and awareness of the condition may be better equipped to provide appropriate care to the growing population of autistic people.^39^

The study participants had suboptimal knowledge of ASD, with a mean score of 55.50% ± 19.70%. A similar study of pharmacists in Palestine had a comparable finding (median score of 50%).^42^ In contrast, similar studies conducted in the US reported higher knowledge scores among pharmacists (84.70% ± 11.70%)^26^; highlighting possible disparities in ASD knowledge between pharmacists in developed and less-developed countries. Participants in the current study were most knowledgeable about the social and behavioral impairments associated with ASD, which aligns with previous studies.^25,26,42,44^ However, we identified several knowledge gaps. Of note, almost half of the participants responded “don’t know” regarding FDA-approved medications for treating irritability associated with ASD and regarding the link between vaccines and ASD. Our findings underscore the need for educational interventions to enhance Nigerian pharmacists’ knowledge of ASD. Given that pharmacists could significantly influence parents’ vaccination decisions,^53^ addressing the vaccine safety knowledge gap is crucial. Notably, most respondents reported not receiving ASD training in pharmacy school nor CE on ASD, which may explain their suboptimal ASD knowledge. Therefore, including ASD in the pharmacy school curriculum and conducting ASD-focused CE for pharmacists may improve pharmacists’ knowledge of ASD. National and state conferences could serve as platforms for such CE programs.

Our results indicate inadequate ASD awareness among respondents, with an overall mean awareness score of 2.90 ± 0.80. This finding is consistent with previous reports of suboptimal awareness of ASD among pharmacists and pharmacy students in other regions.^25,26,42^ Similar to previous studies,^25,26,42^ we found the most awareness deficit regarding community resources to which children exhibiting ASD symptoms may be referred. This is in part reflective of the state of ASD care in Nigeria, as there are limited facilities for the care of autistic people;^47^ however, children exhibiting ASD symptoms may be referred to existing institutions such as psychiatric hospitals and other tertiary hospitals as well as Non-Governmental Organizations (NGOs) that provide ASD care.^47^ Children with ASD in Nigeria tend to present late to clinical settings, with an average age of diagnosis of about 7 years compared to 5 years in the US.^9,54^ Pharmacists, particularly those in community practice, could play significant roles in reducing the late diagnosis of ASD in Nigeria by referring children exhibiting ASD symptoms to the relevant institutions. Strategic national and statewide awareness campaigns are recommended to improve pharmacists’ awareness of ASD. Given the constraints on healthcare resources in Nigeria, it may be necessary to integrate these campaigns with existing mental healthcare awareness initiatives.

Similar to the findings of Khanna and Jariwala,^26^ our results indicate that respondents were moderately confident in their ability to provide ASD medication counseling (3.20 ± 1.20) and moderately comfortable with dispensing medications used to treat ASD (3.60 ± 1.10). While this indicates a baseline level of competence, it raises concerns about the adequacy of pharmacist services provided to autistic people in Nigeria. To improve pharmacists’ confidence and comfortability in providing medication management to autistic people, experiential training on ASD during pharmacy training and hands-on CE programs are recommended. A recent article by Kadi et al^39^ provided guidelines to assist pharmacists in delivering optimal care services to autistic people.

Bivariate analyses showed that community pharmacists had significantly higher knowledge scores than hospital pharmacists, while awareness did not differ between the 2 groups. Furthermore, hospital pharmacists were more confident in their ability to provide ASD medication counseling and felt more comfortable dispensing medications used to treat ASD compared to community pharmacists. It could be that community pharmacists had higher knowledge due to a higher proportion of them receiving ASD training during their pharmacy school as compared to hospital pharmacists. On the other hand, hospital pharmacists’ may have had higher levels of confidence and comfort in medication management of ASD due to their longer practice experience compared to community pharmacists. Nonetheless, the overall knowledge, awareness, confidence, and comfort in medication management of ASD were suboptimal. The observed differences in knowledge and perception between community and hospital pharmacists highlight the need for tailored educational interventions to enhance pharmacists’ ability to care for autistic people in Nigeria. Despite various knowledge and awareness gaps about ASD, it is encouraging that most participants strongly believed in the benefits of ASD training programs and CE as well as the benefits of pharmaceutical care services for autistic people in Nigeria. This indicates a profound desire to contribute actively to the care of autistic individuals and shows the potential for Nigerian pharmacists to play a significant role in ASD care if they receive appropriate training.

We observed a strong correlation between perceptions regarding benefits of ASD CE and benefits of pharmacy services to autistic people in Nigeria. This suggests that pharmacists who perceive the benefits of pharmacist services to autistic people may be more inclined to receive CE on ASD. Additionally, there was a positive correlation between pharmacists’ knowledge scores and their awareness scores, as well as between their awareness scores and confidence levels in providing medication counseling to parents of autistic people. Therefore, it could be inferred that interventions that improve pharmacists’ awareness of ASD may improve knowledge of ASD, as well as their confidence in providing medication counseling and comfort level with dispensing medications used to treat ASD.

It would not be uncommon for individuals who pursue careers in hospital versus community pharmacy to have different demographic and practice-related characteristics. While we showed these differences in the bivariate analyses, we also employed multivariate analyses to control for these confounders. The results showed that having less than 5 years of practice experience, receiving ASD training during pharmacy school, undergoing CE on ASD, and having higher perceptions regarding the benefit of pharmacy services to autistic people in Nigeria were significant positive predictors of ASD awareness. The findings suggest that recent pharmacy graduates are more aware of ASD compared to their more experienced counterparts, which is an encouraging trend for the care of autistic people in Nigeria. Notably, the results contradict the bivariate analysis, which found no significant difference between community and hospital pharmacists’ awareness of ASD. The contradicting results may be attributed to the confounders controlled for in the regression model. However, the observed difference may not be practically significant as both hospital and community pharmacists had suboptimal awareness. Findings from the multivariate analysis further emphasize the importance of undergoing ASD training in pharmacy school and CE on ASD in enhancing awareness of the condition.

## Limitations

Study findings should be interpreted considering certain limitations. Firstly, the inability to perform precise sample size calculations due to the absence of available data on the population sizes of hospital and community pharmacists may limit generalizability. Additionally, the comparison between hospital pharmacists and community pharmacists should be interpreted cautiously, as it involved community pharmacists solely in Lagos state and hospital pharmacists from various states, limiting the generalizability of the findings to the entire country. Furthermore, the self-reporting of ASD awareness and perceptions by pharmacists may have been subject to social desirability bias, but this may have been diminished through survey anonymity. Moreover, recall bias may have influenced the pharmacists’ responses regarding undergoing training on ASD during pharmacy training or CE focused on ASD. Study findings may also have been impacted by selection bias as individuals who attend conferences may be more inclined toward CE, potentially influencing the hospital pharmacists’ knowledge and perceptions of ASD. Additionally, the study scope is confined to hospital pharmacists affiliated with AHAPN, excluding those in private settings who are not affiliated with AHAPN. Nonetheless, to our knowledge, this study is the first to assess Nigerian pharmacists’ knowledge, awareness, and perceptions of ASD. It is also the first known study to compare knowledge, awareness, and perceptions between hospital and community pharmacists. The results could inform future studies and interventions to improve the knowledge and awareness of ASD among Nigerian pharmacists. We recommend future studies on a nationally representative sample of hospital and community pharmacists to provide a more comprehensive and comparable understanding of ASD knowledge, awareness, and perceptions.

## Conclusions

This study revealed that Nigerian hospital and community pharmacists have suboptimal knowledge and awareness of ASD, and limited confidence in providing medication management to autistic people. While community pharmacists had significantly higher knowledge, hospital pharmacists were more confident in their abilities to provide medication management to autistic people. Higher awareness of ASD was associated with being a hospital pharmacist, having higher knowledge of ASD, less practice experience, undergoing ASD training in pharmacy school, and ASD CE. Despite the identified gaps, pharmacist expressed interest in improving their understanding of ASD. Strategic and tailored educational interventions such as including ASD in pharmacy school curricula and providing ASD CE are crucial to equip Nigerian pharmacists for optimal care of autistic people.

## Supplemental Material

sj-docx-1-his-10.1177_11786329241299314 – Supplemental material for Nigerian Hospital and Community Pharmacists’ Knowledge, Awareness, and Perceptions of Autism Spectrum Disorders Supplemental material, sj-docx-1-his-10.1177_11786329241299314 for Nigerian Hospital and Community Pharmacists’ Knowledge, Awareness, and Perceptions of Autism Spectrum Disorders by Ayobami Adesoji Aiyeolemi, Ogochukwu Ukamaka Amaeze, Veronica Okugbeni, Oyinlade Kehinde, Adekunle Faid Adeleke and Jamie C Barner in Health Services Insights
