# Supplementary material for: Nigerian Hospital and Community Pharmacists’ Knowledge, Awareness, and Perceptions of Autism Spectrum Disorders
Source: Health Serv Insights. 2024 Nov 12;17:11786329241299314. doi: 10.1177/11786329241299314 (PMC11555742; doi:10.1177/11786329241299314)
Supplement: sj-docx-1-his-10.1177_11786329241299314 – Supplemental material for Nigerian Hospital and Community Pharmacists’ Knowledge, Awareness, and Perceptions of Autism Spectrum Disorders [file sj-docx-1-his-10.1177_11786329241299314.docx]

**STUDY QUESTIONNAIRE**

**SECTION 1: DEMOGRAPHIC AND PRACTICE DETAILS**

i. Which category below describes your age group?

1. < 25 [ ] 2. 26 - 35 [ ] 3. 36 - 45 [ ] 4. 46 -55 [ ] 5. ˃ 50 [ ]

ii. What is your gender identity?

1.Male [ ] 2.Female [ ]

iii. What is your highest educational qualification?

1. B.Pharm [ ] 2.Msc / Mpharm [ ] 3. PharmD [ ] 4. Ph.D. [ ] 5. WAPCP [ ]

iv. For how long have you been a practicing pharmacist?

1. < 5 years [ ] 2. 5-10 years[ ] 3. 11-15 years [ ] 4. 16-20 years [ ] 5. >20 years [ ]

v. Did you have course(s) or lectures on autism spectrum disorder during your pharmacy degree program

1. Yes [ ] 2. No [ ]

vi. Have you had any continuing education (CE) programs on autism spectrum disorder

1. Yes [ ] 2. No [ ]

*B.Pharm = Bachelor of Pharmacy; Msc/MPharm = Master of Science/ Master of Pharmacy; WAPCP: West African Postgraduate College of Pharmacists*

**SECTION 2:** **PHARMACISTS’ KNOWLEDGE OF ETIOLOGY, PREVALENCE, AND TREATMENT OF AUTISM SPECTRUM DISORDER (ASD)**

1. ASD is a developmental disorder.

1. TRUE [ ] 2. FALSE [ ]

2. Children with ASD have impairments in social interaction, communication or language, and behavioral development.

1. TRUE [ ] 2. FALSE [ ]

3. ASD occurs more commonly among males than females.

1. TRUE [ ] 2. FALSE [ ]

4. ASD is more prevalent than juvenile diabetes.

1. TRUE [ ] 2. FALSE [ ]

5. ASD is more prevalent than Down syndrome.

1. TRUE [ ] 2. FALSE [ ]

6. ASD is curable.

1. TRUE [ ] 2. FALSE [ ]

7. Risperidone and aripiprazole have been approved by the FDA for the treatment of irritability associated with ASD.

1. TRUE [ ] 2. FALSE [ ]

8. Vaccines can cause ASD.

1.TRUE [ ] 2. FALSE [ ]

9. ASD exists only in childhood.

1. TRUE [ ] 2. FALSE [ ]

10. ASD is caused because of emotionally distant, rejecting parents.

1. TRUE [ ] 2. FALSE [ ]

11. Genetic factors play a major role in the etiology of ASD.

1. TRUE [ ] 2. FALSE [ ]

12. ASD is a rare disorder.

1. TRUE 2. FALSE [ ]

**SECTION 2: PHARMACISTS’ AWARENESS OF ASD**

|  | Are you familiar with: | Not familiar at all | Not familiar | Somewhat familiar | Familiar | Completely familiar |
| --- | --- | --- | --- | --- | --- | --- |
| i. | The different symptoms of ASD? |  |  |  |  |  |
| ii. | The different classes of 　　medications 　　　(e.g. 　　　 　antipsychotics, antidepressants, stimulants) that are used in treating the symptoms of ASD? |  |  |  |  |  |
| iii. | The specific behaviors associated with ASD that medications seek to alleviate (e.g. hyperactivity, obsessive compulsive disorder, and self-injury) |  |  |  |  |  |
| iv. | The various side effects produced by medications used in the treatment of ASD symptoms (e.g. sedation, irritation, extrapyramidal symptoms)? |  |  |  |  |  |
| v. | How to help families sort through information to make informed decisions about their child with ASD? |  |  |  |  |  |
| vi. | Community resources in your region that can be used for referral of a child who is exhibiting symptoms commonly associated with ASD? |  |  |  |  |  |

**SECTION 4:** **TRAINING AND CONFIDENCE OF PHARMACISTS IN MEDICATION MANAGEMENT OF ASD**

|  |  | Strongly disagree | Disagree | Neutral | Agree | Strongly agree |
| --- | --- | --- | --- | --- | --- | --- |
| i. | I feel confident in my ability to counsel parents about the medication profile and side effects of prescriptions being used for the treatment of their child with ASD |  |  |  |  |  |
| ii. | I feel comfortable dispensing medications used in the treatment of ASD |  |  |  |  |  |
| iii. | I feel that I could benefit from taking a continuing education or training program in the area of ASD |  |  |  |  |  |
| iv. | I feel that pharmacy school curriculum should include a course or lecture in the area of ASD |  |  |  |  |  |
